# Parvovirus B19 in Children: Clinical Spectrum, Viral Load Patterns, and Atypical Cutaneous Presentations in the Post-Pandemic Outbreak

**DOI:** 10.3390/v18020223

**Published:** 2026-02-10

**Authors:** Sanda Škrbina, Dominik Ljubas, Ivana Valenčak, Leo Markovinović, Oktavija Đaković Rode, Snježana Zidovec-Lepej, Goran Tešović

**Affiliations:** 1 Pediatric Infectious Disease Department, University Hospital for Infectious Diseases ‘Dr. Fran Mihaljević’, Mirogojska 8, 10000 Zagreb, Croatia; sskrbina@bfm.hr (S.Š.); dljubas@bfm.hr (D.L.);; 2 School of Medicine, University of Zagreb, Šalata 3, 10000 Zagreb, Croatia; 3 Department of Virology, University Hospital for Infectious Diseases “Dr. Fran Mihaljević”, Mirogojska 8, 10000 Zagreb, Croatia; 4 School of Dental Medicine, University of Zagreb, Gundulićeva 5, 10000 Zagreb, Croatia; 5 Department of Immunological and Molecular Diagnostics, University Hospital for Infectious Diseases ‘Dr. Fran Mihaljević’, Mirogojska 8, 10000 Zagreb, Croatia

**Keywords:** human parvovirus B19, pediatrics, petechiae, PAPPE, erythema infectiosum

## Abstract

**Background:** Human parvovirus B19 causes a broad spectrum of clinical manifestations, ranging from the classic “fifth disease” to severe presentations. Clinical presentation varies considerably across age groups. In 2023–2024, a notable increase in parvovirus B19 cases was reported across Europe. **Methods:** We retrospectively analyzed pediatric patients with serum serology and/or plasma PCR-confirmed parvovirus B19 infection treated at the tertiary infectious diseases center (University Hospital for Infectious Diseases, Zagreb) in 2023 (January–August). Demographic, laboratory, viral load, and clinical characteristics were assessed, with emphasis on cutaneous manifestations. **Results:** A total of 102 patients were included (median age 10 years; 54.9% male), of whom 7.8% required hospitalization. Rash was present in 94 (92.2%) of the patients of whom 75 had erythema infectiosum and petechiae, while the rest had a combination of both. Patients with petechial rash were significantly older (*p* = 0.013) and exhibited lower platelet counts (*p* < 0.001) compared with those with erythema. A higher proportion of anti-B19V IgM (*p* = 0.027) and IgG (*p* < 0.001) antibodies was detected in patients with erythema. Petechial rash was associated with higher viral loads (*p* < 0.001). In univariate analysis, the presence of anti-B19V IgG antibodies was correlated with the absence of petechial rash (OR = 0.09; *p* < 0.001), whereas higher viral load was associated with its presence (OR = 1.7; *p* < 0.001). In multivariate analysis, viral load emerged as the only predictor of petechial rash (aOR = 1.4, *p* = 0.042). **Conclusions:** Parvovirus B19 remains a self-limiting illness in healthy children, despite frequent atypical presentations. Higher viremia is associated with atypical rash morphology and suggests age-related differences in immune clearance.

## 1. Introduction

Human parvovirus B19 (B19V) is a single-stranded DNA virus belonging to the family *Parvoviridae*, first identified in 1975 [1,2]. B19V is a globally widespread virus, predominantly affecting school-aged children. It typically exhibits a cyclical pattern of increased circulation, causing smaller outbreaks every 3–4 years, typically in late winter and early spring [2]. The primary routes of B19V transmission are via saliva, respiratory secretions, or infected blood products [3].

The clinical presentation of B19V infection ranges from asymptomatic or mild disease, to severe, life-threatening complications in high-risk groups, including immunocompromised individuals and patients with underlying hematological disorders [3,4]. In children, erythema infectiosum (EI) is the most common clinical manifestation of B19V infection [2,5]. The disease usually presents as a biphasic illness, with a prodromal phase that includes constitutional symptoms, followed by erythema, and sometimes arthralgias [2,5]. B19V infection may also present with papular-purpuric ‘gloves and socks’ syndrome (PPGSS), as well as various non-classical exanthems [6,7]. During the viremic phase, B19V may cause transient hematologic changes that usually resolve spontaneously. However, patients with underlying hematological disorders are at risk of severe anemia, including transient aplastic crisis and pure red-cell aplasia (PRCA) [8].

The clinical spectrum of B19V reflects the host immune response to the pathogen. Certain manifestations, such as anemia, are considered to be a consequence of the direct cytopathic effect, while rash and arthralgia are considered to be immune-mediated features [2,9,10]. However, existing literature offers only limited evidence of a possible relationship between viremia, antibody levels, and the severity of clinical manifestations. So far, those correlations have only been noted in the context of viremia and severity of anemia, mostly as brief comments or observations [11,12,13]. Certain evidence suggests that petechial skin lesions correspond to leukocytoclastic vasculitis mediated by antibody-dependent cellular immunity [7]. Conversely, in cases of PPGSS B19V, antibodies are usually absent, while viral DNA might be detected, indicating that skin manifestations might be also related to direct viral cytopathic effect [14].

The European Center for Disease Prevention and Control (ECDC) reported a significant increase in B19V infections across several European Union/European Economic Area (EU/EEA) countries in 2023/2024 [15]. Recent studies from Israel, France, Italy, Denmark, and the Netherlands have documented a notable resurgence of B19V infections [16,17,18,19,20].

The aim of this study is to describe demographic, clinical, and laboratory features associated with acute B19V infection among pediatric patients treated at the University Hospital for Infectious Diseases ‘Dr. Fran Mihaljević’ (UHID) during a B19V outbreak that occurred from January to August 2023. Given the aforementioned distinct pathophysiological mechanisms underlying skin involvement, we attempted to investigate the correlation between viral loads, antibody titers, and different skin manifestations of the infection.

## 2. Materials and Methods

### 2.1. Patients and Related Clinical Data

We retrospectively analyzed the B19V outbreak among pediatric patients who were admitted to UHID. We included patients who were treated as both inpatients and outpatients. The outbreak lasted from January to August 2023. Eligibility for inclusion were patients aged 0 to 18 years who presented to our emergency department with symptoms, as well as clinical and laboratory findings associated with acute B19V infection. In all included patients, B19V infection was proven using either serological or molecular diagnostic assays, or by a combination of both.

We reported demographic data, including age and gender, along with laboratory and clinical findings associated with the infection. Additionally, we collected medical data regarding other viral and bacterial co-infections that were detected along with the acute B19V infection.

Reported laboratory data included blood count parameters, including leukocyte count (× 10^9^/L), hemoglobin (Hgb) levels (g/L), and platelet count (× 10^9^/L). C-reactive protein (CRP) values (mg/L) and alanine aminostransferase (ALT) levels (IU/L) were also reported. In patients presenting with petechial rash, markers of coagulation (prothrombin time, fibrinogen levels, activated partial thromboplastin time, thrombin time) were assessed in all patients, and were within the reference ranges.

Clinical findings included fever, headache, abdominal pain, arthralgias, and cutaneous manifestations. For further statistical analysis, we categorized the patients based on rash morphology and B19V-related serological and molecular findings.

### 2.2. Detection of B19V and Additional Microbiological Testing

Acute B19V infection was defined based on the analysis of serological and molecular diagnostic tests in patients who had clinical and laboratory findings attributable to B19V. Serum anti-B19V IgM and IgG antibodies were qualitatively measured using a chemiluminescent immunoassay (CLIA), *LIAISON^®^* Biotrin Parvovirus B19 for IgM and IgG (DiaSorin S.p.A., Saluggia, Italy) on the *LIAISON^®^ XL* analyzer (DiaSorin S.p.A., Saluggia, Italy). The analyzer qualitatively measures anti-B19V antibody levels, expressed as Index values, with values equal to or greater than 1.10 suggesting B19V exposure.

Quantification of B19V DNA in the plasma was performed using a standardized quantitative real-time PCR assay (*AltoStar^®^ Parvovirus B19 PCR Kit 1.5* (Altona Diagnostics, Hamburg, Germany) on the *LightCycler^®^ 480 Instrument* (Roche Diagnostics, Pleasanton CA, USA), according to the manufacturer’s instructions. The limit of detection in plasma is 121 IU/mL (95% CI ranging from 79 to 218 IU/mL), with a validated linear range for quantification from approximately 500 to 1 × 10^8^ IU/mL. Values below the quantification range were considered detectable but not quantifiable.

In 96 (94.1%) and 79 (77.5%) of patients, serum and plasma samples were collected for serological and PCR assays, respectively. In six patients (5.9%), only the PCR assay was performed, while in 23 patients (22.5%), only serological assays were carried out. In these patients, anti-B19V IgM or both anti-B19V IgM and IgG were detected. In the remaining patients (73; 71.6%), both serological and molecular assays were performed. In all except three patients, serum and plasma samples were collected on the same day. In the latter group, 10 out of 73 patients had negative anti-B19V IgM antibodies, with a positive PCR assay (Table 1). Among the remaining patients (63 out of 73), both anti-B19V antibodies and B19V DNA were detected. We reported viral load values (IU/mL) and qualitative measurements of anti-B19V IgM and IgG, together with the timing of sample collection.

Additional microbiological testing was performed according to clinical features, and included throat swabs for bacterial culture, nasopharyngeal swabs for the detection of respiratory pathogens using a multiplex PCR panel (*Respiratory-well 16*, AusDiagnostics, Sydney, Australia), along with blood cultures and serum samples for serological assays. Serological assays were used to exclude alternative causes. As the majority of our patients presented with atypical skin manifestations or various degrees of bicytopenia, we employed serological assays to exclude infectious agents that represent the main differential diagnosis based on these features (*Cytomegalovirus, Epstein-Barr virus, Mycoplasma pneumoniae, Chlamydophila pneumoniae, Chlamydia psittaci, Human herpes virus 6, Anaplasma species*). The PCR multiplex respiratory panel detects the following viruses: influenza A and B, respiratory syncytial virus A and B, human parechovirus, enterovirus, adenovirus, rhinovirus, bocavirus, SARS-CoV-2 and seasonal coronaviruses, and human parainfluenza viruses 1–4. Additional microbiological workup was not uniform in all patients and was guided by clinical judgment.

### 2.3. Statistical Analysis

Numerical variables were reported as medians and means, while absolute and relative frequencies were calculated for categorical variables. The normality of distribution of continuous variables was assessed graphically and with the Shapiro–Wilk test. All numerical variables were non-normally distributed (Shapiro–Wilk test, *p* < 0.05). Therefore, for comparison of continuous variables, the Mann–Whitney U or the Kruskal–Wallis tests were employed, as applicable. For categorical variables, associations were assessed using chi-square or Fisher’s exact tests, as appropriate.

We compared rash morphology in relation to age, gender, days of rash onset, laboratory findings, viral load levels, and serological findings (Table 2). As sampling time for both serum and plasma specimens was uniform for almost all cases, we also investigated the relationship between IgM and IgG serostatus in relation to viral loads, Hgb levels, and platelet count. A pairwise Wilcoxon rank-sum test with Benjamini–Hochberg correction for multiple comparisons was used to compare viral load, hemoglobin levels, and platelet count medians between groups with different serostatus.

Furthermore, to explore factors potentially associated with petechial exanthem, bivariate and multivariable logistic regression models were calculated. Age in years, gender, and time interval between rash onset and sampling were included as potential confounders, together with anti-B19V IgM and IgG seropositivity and log_10_-transformed viremia levels (Table 3). Categorical variables, including gender, IgM, and IgG seropositivity were dummy coded, while other variables were used as continuous variables. Variables that were statistically significant in univariate analysis were used in the multivariate model. Collinearity between variables was assessed using variance inflation factors.

All statistical tests were two-tailed with the significance level set to 0.05. Data were analyzed using R version 4.5.1 (R Development Core Team, Vienna, Austria).

### 2.4. Ethical Approval

The study was approved by the UHID Ethical Committee (Approval no. 01-2745-3-2025). No identifiable patient data were disclosed.

## 3. Results

### 3.1. Baseline Characteristics, Cutaneous and Other Clinical Manifestations

A total of 102 children with confirmed B19V infection were included in the study, of whom 56 (54.9%) were male. The median age was 10 years. Most of the patients were older than 5 years (89.2%). Three patients had significant hematological comorbidities: one was diagnosed with spherocytosis, one with thalassemia, and one with mutation of the beta-globin gene (Köln mutation). The remaining patients were otherwise healthy. Eight patients (7.8%) were hospitalized. The outbreak lasted from January to August 2023. A positive epidemiological link was detected in 17 out of 102 patients (16.7%), of whom nine had probable household exposure.

**Figure 1:**
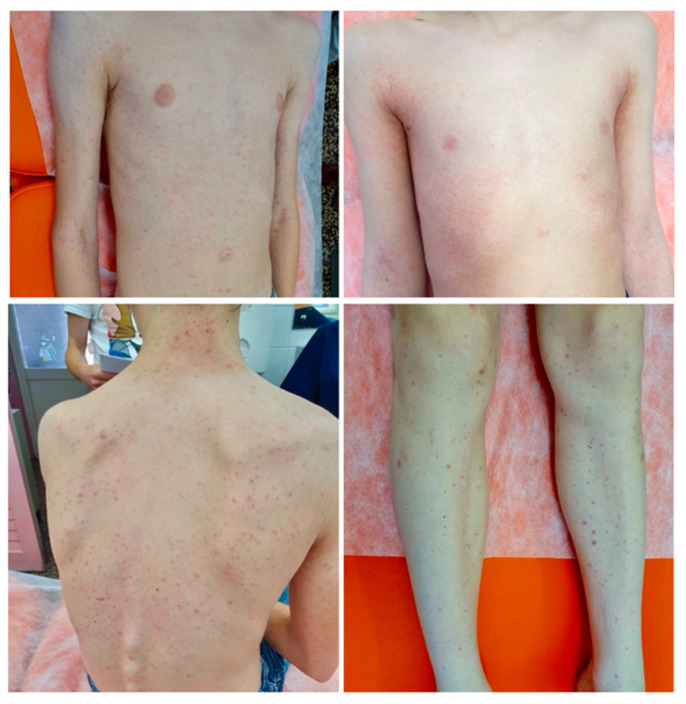
Petechial rash equally distributed on trunk and extremities.

The demographic and clinical characteristics of our patients are presented in Table 1. Rash was the most common symptom, occurring in 94 (92.2%) of patients. Among patients exhibiting a rash, 43 (45.7%) had a typical rash resembling EI. It is noteworthy that a high proportion of patients (32, 34.0%) presented with petechial or purpuric rash (Figure 1). In total, 66 patients (64.7%) had fever, with an average duration of 3.8 days. Almost three quarters of patients (47, 71.2%) had fever prior to the onset of rash. Eight patients (9.8%) had no rash, and 10 presented with both EI and petechiae (10.6%). Finally, 9 (9.6%) patients developed a rash that could not be classified as either EI or petechiae. Only a minority of patients with a rash (22, 22.9%) had itching. In addition to rash, the most prevalent symptoms in our cohort were headache (23, 22.5%), cough (18, 17.6%), and abdominal pain (13, 12.7%). Interestingly, arthralgias were noted in only 11 (10.8%) patients.

### 3.2. Microbiological Patterns Associated with Acute B19V Infection

In all patients, the infection was confirmed by either targeted PCR or a serological assay. In total, anti-B19V IgM and IgG assays were performed in 96 (94.1%) of patients, and a targeted PCR assay was performed in 79 (77.5%) of patients. Among the patients in whom serology was obtained, 86 (89.6%) had positive anti-B19V IgM antibodies, with anti-B19V IgG detectable in 50 cases concurrently. In all children in whom the targeted PCR assay was performed, viremia was detected. Based on microbiological findings associated with acute B19V infection, four groups of patients were defined. The first group consisted of patients with a positive anti-B19V IgM or both anti-B19V IgM and IgG, in whom the diagnosis was established by a serological assay alone (23, 22.5%). The second group included patients in whom only the PCR assay was performed and who had B19V DNA detected in plasma (6, 5.9%). The third group comprised patients in whom diagnosis was made by the detection of B19V DNA in plasma along with positive anti-B19V IgM or both IgM and IgG (63, 61.8%). The fourth group included patients in whom B19V DNA was detected in plasma; serological testing was performed, but was negative for anti-B19V IgM antibodies (10, 9.8%). However, in the latter group, in three patients anti-B19V IgG was detected. 

**Figure 2:**
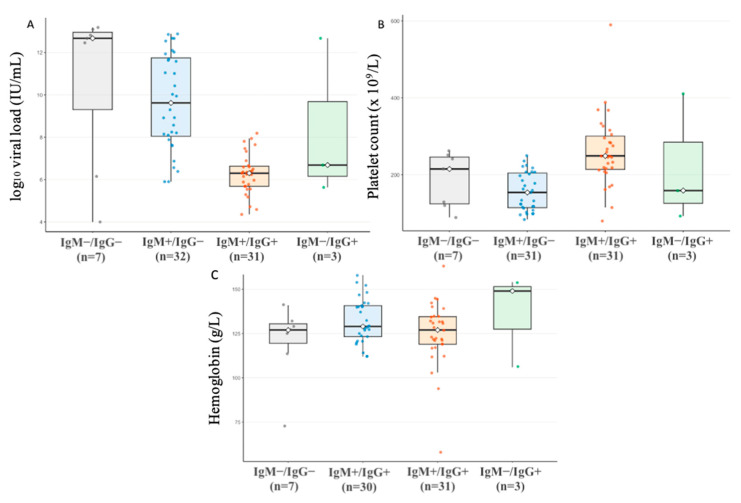
Differences in viral load (expressed as log_10_ IU/mL) (**A**), platelet count (**B**), and Hgb levels (**C**) according to anti-B19V IgM and IgG detection. Patients with missing values were excluded. Individual values are represented with jittered points. Boxes represent interquartile ranges. Medians are indicated by horizontal lines.

Figure 2 represents the comparison of B19V DNA levels (expressed as log_10_ IU/mL), platelet counts, and Hgb levels across different serostatus groups. We identified four different groups of patients based on serological findings. In this comparison, we included only the 73 patients in whom both PCR and serology assays were performed. The highest viral loads were observed in the group with negative serological findings, while the lowest viral loads were noted among patients who developed both IgM and IgG antibody response. Post hoc pairwise comparison showed that median viral load values were significantly higher among patients with positive anti-B19V IgM compared with those who had both anti-B19V IgM and IgG antibodies (9.6 log_10_ IU/mL, IQR 8.0–11.7 vs. 6.3 log_10_ IU/mL, IQR 5.7–6.6, *p* < 0.001). Platelet count was lower among those who were only anti-B19V-IgM positive compared to those in whom both anti-B19V-IgM and IgG were detected (154 × 10^9^/L vs. 249 × 10^9^/L, *p* < 0.001). For Hgb levels, no differences were found (Figure 2).

### 3.3. Laboratory and Microbiological Findings

Average leukocyte count in our patients was 6.8 × 10^9^/L (median 5.9 × 10^9^/L), and average CRP level was 21.0 mg/L (median of 5.6 mg/L). Most patients exhibited low CRP levels; higher CRP values were predominantly observed in patients with confirmed co-infection. Only three patients (2.9%) had Hgb levels lower than 100 g/L, and all three had hematological comorbidities. Among them, a 6-year-old male patient with spherocytosis developed severe anemia (Hgb 58 g/L) and required an erythrocyte transfusion. Thrombocytopenia was an infrequent feature of the infection, with a platelet count lower than 150 × 10^9^/L found in around one-quarter of patients (26/102, 26.0%). None of the patients exhibited severe thrombocytopenia (platelet count lower than 50 × 10^9^/L). Elevated ALT levels (>50 U/L) were observed in 9 patients (8.8%), with a median value of 35.2 U/L. However, none of these patients exhibited clinical signs of acute hepatitis (Table 2).

Co-infection was detected in 20 (19.6%) patients, with viral co-infection being the most common. Adenovirus was detected in six patients, rhinovirus in two, and one patient had adenovirus, rhinovirus, enterovirus, and human parainfluenza virus 3 detected from a nasopharyngeal swab. Two patients had clinically diagnosed acute VZV infection. In addition, acute Epstein–Barr virus infection was serologically confirmed in two cases. Bacterial co-infections included *S. pyogenes*, detected from throat swabs in seven cases, and urinary tract infection caused by *E. coli* in two patients. Two patients had acute gastroenteritis, caused by *C. jejuni* and *C. difficile*, respectively. One patient had radiologically confirmed pneumonia and one patient had acute unilateral cervical lymphadenitis confirmed by ultrasound. All other additional microbiological tests, including serological assays for alternative causes were negative.

To assess CRP as a potential marker of co-infection, multivariate logistic regression analysis was used with CRP values, age, and gender as independent variables. Based on the analysis, increasing age was associated with reduced odds of co-infection (OR = 0.77; 95% CI: 0.64–0.91; *p* = 0.003), while higher CRP levels were associated with an increased risk of co-infection (OR = 1.03; 95% CI: 1.01–1.06; *p* = 0.003).

**Table 2:** Laboratory and microbiological parameters among patient groups stratified by rash morphology.

	Total	Only EI Rash (Group 1)	Only Petechial Rash (Group 2)	Both Exanthems (Group 3)	*p* **
Number of patients	102 (100.0)	43 (42.2)	32 (31.4)	10 (9.8)	
Male	56 (54.9)	19 (44.1)	18 (56.2)	8 (80.0)	0.424 ^†^
Age (months)					
Mean (median)	118.7 (120.5)	105.9 (111.6)	132.0 (127.8)	126.3 (120.0)	0.013 ^§^
Rash onset (days)					
Mean (median)	2.9 (2.0)	2.6 (1.0)	2.6 (2.0)	2.9 (1.5)	0.318 ^§^
Leukocyte count (×10 ^9^/L) Mean (median) Minimum; Maximum value	6.8 (5.9) 1.6; 19.9	7.7 (7.2) 1.7; 18	5.5 (4.4) 1.7; 19.9	6.1 (5.7) 1.6; 15.0	0.002 ^§^
Platelet count (×10 ^9^/L) Mean (median) Minimum; Maximum value	221 (214) 80; 590	259 (249) 115; 589	171 (173) 84; 305	185.3 (143) 113; 327	<0.001 ^§^
Hgb (g/L) Mean (median) Minimum; Maximum	128 (129) 58; 163	128.8 (131) 58; 163	128.5 (128) 107; 158	130.7 (127) 109; 159	0.473 ^§^
CRP (mg/L)					
Mean (median)	21.0 (5.4)	11.8 (1.45)	25.9 (7.9)	17.7 (3.6)	<0.001 ^§^
ALT (U/L)					
Mean (median)	35.2 (18.0)	40.4 (36.0)	33.8 (19.0)	16.0 (14.5)	0.137 ^§^
Serology performed	96 pts	42 pts	30 pts	8 pts	-
anti-B19V IgM positivity rate * (%)	86 (89.6)	42 (100.0)	26 (86.7)	7 (87.5)	0.027 ^†^
anti-B19V IgG positivity rate * (%)	53 (55.2)	35 (83.3)	7 (26.9)	3 (42.9)	<0.001 ^†^
Viral load (IU/mL) Mean Median	9.9 × 10^11^ 2.2 × 10^7^	3.1 × 10^7^ 2.3 × 10^6^	2.0 × 10^12^ 2.7 × 10^10^	6.6 × 10^10^ 2.6 × 10^7^	<0.001 ^§^

ALT—alanine aminotransferase; B19V—Parvovirus B19; CRP—C-reactive protein; EI—erythema infectiosum; Hgb—hemoglobin; pts—patients. * Among those in whom serology was performed; ** Comparison between Group 1 and 2. ^†^ Fisher exact test/chi-square test; ^§^ Wilcoxon rank-sum test.

### 3.4. Laboratory and Diagnostic Comparison Across Rash Phenotypes

Patients were classified into three groups based on rash morphology: those with only EI (Group 1), those presenting with only petechial exanthems (Group 2), and those exhibiting both types of rashes (Group 3). We compared rash morphology in relation to age, gender, days of rash onset, laboratory findings, viral load levels, and serological findings between Group 1 and Group 2 (Table 2). In this analysis, we excluded patients who had combined skin lesions or other types of rashes due to the small subset of individuals in these groups (10 and 9, respectively).

Patients with petechial rash were significantly older (127.8 vs. 111.6 months, *p* = 0.013), while no difference was found between genders. Moreover, petechial rash was associated with a lower thrombocyte count (173 vs. 249 × 10 ^9^/L, *p* < 0.001) and higher CRP values (7.9 vs. 1.45 mg/L, *p* < 0.001). A higher proportion of anti-B19V IgM (100.0% vs. 86.7%, *p* = 0.027) and IgG (83.3% vs. 26.9%, *p* < 0.001) antibodies was detected among children with an EI-type rash. Conversely, patients with petechial exanthem had a higher median viral load compared with the EI group (10.4 log_10_ IU/mL, IQR: 7.8–12.7 vs. 6.3 log_10_ IU/mL, IQR: 5.7–7.3, *p* < 0.001).

The results of univariate and multivariate analyses are presented in Table 3. In univariate analysis, the presence of anti-B19V IgG antibodies (OR = 0.09; *p* < 0.001) and longer time interval between rash onset and sample collection (OR = 0.75, *p* = 0.043) were associated with the absence of petechial rash. Moreover, a 10-fold increase in viral load was highly associated with a petechial rash (OR = 1.7, *p* < 0.001). In multivariate analysis, viral load remained the only significant variable independently associated with a petechial rash (aOR = 1.4, 95% CI: 1.03–2.01, *p* = 0.042), while the time interval between rash onset and sampling was not significantly associated with antibody presence.

**Table 3:** Univariate and multivariate regression analysis of factors associated with petechial exanthem.

Variable (*)	OR (95% CI)	*p*	aOR (95% CI)	*p*
Age in years (per 1 year)	**1.21 (1.07–1.39)**	**0.005**	1.19 (0.96–1.55)	0.141
Gender (female)	1.89 (0.83–4.39)	0.130	2.13 (0.54–9.28)	0.288
IgG seropositivity (negative)	**0.09 (0.03–0.23)**	**<0.001**	0.27 (0.06–1.33)	0.106
IgM seropositivity (negative)	0.28 (0.04–1.36)	0.136	-	-
Viral load (per 10-fold increase)	**1.70 (1.34–2.31)**	**<0.001**	**1.40 (1.03–2.01)**	**0.042**
Time interval between rash onset and sampling (days)	**0.75 (0.53–0.93)**	**0.043**	0.83 (0.53–1.02)	0.207

* Reference values for categorical variables/units increase for continuous variables. Values that were stastistically significant were bolded.

### 3.5. B19V as a Probable Cause of Polyserositis Syndrome

We observed an interesting case of polyserositis in a 6-year-old, otherwise healthy girl. She initially presented with high-grade fever, generalized lymphadenopathy, and an erythema infectiosum-like rash. Initial laboratory evaluation revealed markedly elevated inflammatory markers (procalcitonin 34.9 ng/mL, CRP 59.4 mg/L), and empiric antibiotic therapy with ceftriaxone was initiated for a total of five days without clinical improvement. Due to persistent fever and sustained elevation of inflammatory markers, an additional diagnostic workup was performed. Polyserositis was confirmed by ultrasound examination, demonstrating a circumferential pericardial effusion measuring 8–9 mm anterior to the right ventricle and approximately 6 mm posterior to the left ventricle, bilateral pleural effusions up to 5 mm at the lung bases, and a small amount of free fluid in the Douglas pouch. Chest radiography showed no pathological findings.

Apart from elevated inflammatory markers, laboratory findings were notable for mild thrombocytopenia (platelet count 136 × 10^9^/L), while the remainder of the extensive laboratory evaluation was within normal limits. During the course of the illness, the patient also developed diarrhea and vomiting.

B19V infection was confirmed by plasma PCR (62,000,000 IU/mL). A serological assay was not performed, based on the clinician’s decision. No co-infections were detected (including throat swab for *S. pyogenes*, urine and stool culture, and EBV serology and plasma PCR). Treatment with indomethacin in combination with gastroprotective therapy resulted in complete regression of the effusions and full clinical recovery. During subsequent follow-up, the patient remained asymptomatic and required no further diagnostic evaluation.

## 4. Discussion

We investigated the demographic, clinical, and laboratory features of the B19V outbreak among pediatric patients in the context of a recent increase in B19V activity recorded across multiple European countries during 2023 and 2024 [17,21,22]. Additionally, we aimed to assess the association between viral burden and cutaneous manifestations of the infection, as evidence so far has suggested that both direct viral and indirect immunological mechanisms are responsible for these manifestations. Except for the outbreak in 2013/14, so far there have been no studies on demographic, laboratory, and clinical features of B19V infection in Croatia [23]. Data on seroprevalence and epidemiology of B19V in Croatia are also scarce: a study from 2010–2024 showed IgM and IgG seropositivity rates of 9.8% and 42.25% among children and adolescents, respectively [24,25].

In the majority of cases in our cohort, the clinical presentation did not differ from the typical clinical course of B19V infection in immunocompetent children, which aligns with reported data on B19V epidemics in other European countries, where the overall risk to the general population was assessed as low [15]. The literature on clinical features of recent B19V outbreaks is limited, as most of these studies were focused solely on seroepidemiology [5,16,17,26]. In a recent Italian outbreak reported in 2024, 204 patients met criteria for primary B19V infection, with a median age of 7.7 years. Hospitalization was required in 73.5% of the cases, including 17 patients who were admitted to the intensive care unit [5]. These findings contrast with our cohort, in which no severe outcomes were observed apart from one severe case of anemia. Compared with the previously reported B19V epidemic among Croatian children in 2013, we found a lower prevalence of joint and liver involvement and anemia, although thrombocytopenia was more frequently observed [23]. In a 2023 French outbreak, rash and fever were also the most common symptoms, but with higher rates of extracutaneous features, including arthralgia and hematologic involvement [27]. Interestingly, an Italian study found a much lower prevalence of cutaneous involvement compared with our data [28]. 

In general, inflammatory markers were low in most children. When elevated, they were usually associated with co-infections, occurring in 19.6% of the cohort and suggesting that co-infections during acute B19V are rare. Moreover, co-infections were more common among younger patients. The majority of co-infections were viral, with no significant clinical repercussions. The aforementioned issue is rarely addressed in the literature, likely because in immunocompetent children B19V usually causes a mild, self-limiting illness that often goes unnoticed. However, in immunocompromised patients viral co-infections might aggravate preexisting cellular damage, especially in the case of viral hepatotropism [29]. Based on our results, in otherwise healthy children presenting with clinical findings consistent with acute B19V, high inflammatory markers should prompt further diagnostic evaluation for other infectious agents.

Although most patients experienced a typical disease course, around one-third of them were febrile with concomitant petechial skin changes (Figure 1). The distribution and morphology of rash did not correspond to PPGSS characterized by sharply demarcated acral purpura [30]. While several focal variants of B19V-related skin eruptions, including PPGSS, acropetechial syndrome, unilateral laterothoracic exanthem, and asymmetric periflexural exanthem have been described, generalized purpuric-petechial eruptions remain an uncommon feature of skin involvement during the infection [6,31,32,33]. The broader concept of parvovirus B19-associated purpuric-petechial eruption (PAPPE) was first proposed by Hashimoto and Yuno in 2011, based on two case reports in pediatric patients [34]. Following the first report of PAPPE, several other case reports and series of B19V have addressed similar findings consistent with the patterns observed in our cohort [35,36,37,38]. In light of the recent B19V epidemics reported in European countries, four cases in Turkey were associated with skin involvement consistent with PAPPE [39]. Atypical cutaneous eruptions are more common in older children, supporting the hypothesis that skin manifestations might be age-related [35,36,37,38,39]. Along with the well-established cytopathic effect of B19V on erythroid progenitors, evidence also suggests that B19V displays a cytotoxic effect on endothelial cells, which provides an explanation for petechial efflorescences [40,41]. In contrast, immune-complex deposition is a strongly supported mechanism for the development of EI during the second phase of illness [42]. In our cohort, patients presenting with the typical EI rash had a higher prevalence of anti-B19V IgM as well as anti-B19V IgG titers compared with the group presenting with petechial rash. Univariate analysis showed that the presence of anti-B19V IgG reduced the likelihood of petechial rash, while in the multivariate model, this effect was less pronounced; instead, viral load emerged as an important independent predictor of petechial rash. Significantly higher viral loads were found in children with petechial-purpuric rash, most of whom were older than five years, which might be attributed to age-related differences in immune response to the pathogen, subsequently leading to differences in the time required for viral clearance. We hypothesize that this finding likely reflects the timing of the host immune response: isolated petechiae correspond to an earlier stage of high viral replication, whereas the appearance of EI marks the transition to the immune-mediated phase.

Our findings are further supported by comparisons of viral load levels across different serological profiles (Figure 2). Given our results, it seems that the emergence of anti-B19V IgG antibodies is pivotal for achieving viral clearance, as these patients exhibited significantly lower viral loads. We did not find any differences in Hgb levels, probably due to the fact that only a few of the patients had underlying hematological disorders. However, a lower platelet count was observed among patients who were only anti-B19V IgM seropositive. This might be explained by the direct effect of B19V on megakaryocytes, which is a well-described viral feature in vitro [43].

In the view of the above, additional studies are required to address immune mechanisms underlying clinical manifestations, especially skin involvement. The correlation between rash type and viral load also questions the level of infectivity following rash development, given that individuals with an atypical rash demonstrate a higher viral burden and a possibly delayed shift to the immune-mediated phase, in contrast to those who develop EI. 

In light of the fact that viral load and antibody titers can significantly vary at different stages of acute B19V infection, it is important to know the time interval between rash onset and sample collection for accurate interpretation of laboratory findings. Paired serology, along with a serial viral load measurement, was not performed in our cohort due to the generally mild nature of the disease, but would be useful in future studies to fully validate the interpretation of these findings. In three patients, an interesting serological profile was observed: only IgG antibodies were detected, accompanied by relatively high viral loads (Figure 2). These patients had documented episodes of recurrent rash prior to the current disease episode. Although IgG seropositivity might be attributable to antibody cross-reactivity, these features raise the possibility of persistent B19V infection, similar to cases reported in adults [44]. In this group, paired serology and longitudinal viral load measurements would be particularly useful for the interpretation of these findings.

While previous reports highlight purpuric-petechial eruptions as an unusual finding, we observed a much higher frequency of atypical skin eruptions. We believe that one of the potential explanations for a higher incidence of petechial rash/PAPPE lies in the ‘immunity gap’ that emerged after the COVID-19 pandemic, as reduced circulation of endemic viruses, including B19V, during prolonged non-pharmaceutical interventions (NPI) resulted in a diminished natural boosting of population immunity [45]. Therefore, we argue that waning immunity may contribute to prolonged viral clearance and enhanced cytopathic effect of the virus during the acute phase of the illness. This might also be related to mutations in B-cell epitopes of viral capsid proteins that might have occurred during the implementation of NPIs, as these are target proteins for neutralizing antibodies and are therefore pivotal for viral clearance and the induction of B-cell memory [46,47]. Genomic surveillance of the B19V outbreak in Italy during 2024 showed the G1a genotype to be a circulating genotype with detected mutations in viral capsid proteins, although their impact on host immune response still needs to be evaluated [18]. Recent studies in other European countries, including France and Switzerland, as well as preliminary genotyping data from the Croatian 2023 cohort, confirm the continuous circulation of B19V genotype 1a in various European regions [27,48,49,50]. Therefore, comprehensive phylogenetic and molecular surveillance of B19V genotype 1 should be highly encouraged in the future to explore potential clinical effects of such mutations. 

## 5. Conclusions

This is the first retrospective study of the 2023 B19V outbreak in pediatric patients in Croatia. Despite the ‘immunity debt’ caused by COVID-19, B19V is still a self-limited illness, with rare complications or co-infections in previously healthy children. 

Cutaneous manifestations associated with B19V have shown an evolving trend during the post-pandemic period, with an increased frequency of atypical rashes resembling PAPPE, mostly among older children. Therefore, B19V should be suspected in otherwise healthy, well-appearing children presenting with fever and petechial skin changes. Based on our observations, immune mechanisms underlying viral clearance might be age-dependent and warrant further studies.

Several limitations must be acknowledged. As most of the patients in our cohort had a self-limiting illness presenting with a clinically recognizable rash, paired serology samples were not obtained. For the same reason, no follow-up was conducted. However, obtaining paired sera as well as serial viral load measurements would provide a better insight into the disease course and underlying pathophysiological mechanisms. Moreover, phylogenetic analysis and genotyping of viral isolates would provide more comprehensive insights and contribute relevant evidence to post-pandemic molecular and epidemiological surveillance of B19V in Europe. The absence of such data constitutes the main limitation of our study and warrants further investigation.

## Figures and Tables

**Table 1:** Demographic and clinical characteristics among groups according to B19V serostatus and PCR.

	Total	Only IgM+/IgG− or IgM+/IgG+	Only PCR+	IgM+/IgG− or IgM+/IgG+ and PCR+	IgM−/IgG− or IgM−/IgG+ and PCR+
Number of patients (%)	102 (100.0)	23 (22.5)	6 (5.9)	63 (61.8)	10 (9.8)
Gender					
Male	56 (54.9)	13 (56.5)	3 (50.0)	35 (55.6)	5 (50.0)
Female	46 (45.1)	10 (43.5)	3 (50.0)	28 (44.4)	5 (50.0)
Age (years)					
0–5	11 (10.8)	4 (17.4)	1 (16.7)	5 (7.9)	1 (10.0)
6–18	91 (89.2)	19 (82.6)	5 (83.3)	58 (92.1)	9 (90.0)
Rash *	94 (92.2)	21 (91.3)	6 (100.0)	60 (95.2)	7 (70.0)
EI	43 (45.7)	14 (66.7)	1 (16.7)	28 (46.7)	0(0.00)
Petechial	32 (34.0)	3 (14.4)	2 (33.3)	23 (38.3)	4 (57.4)
Fever	66 (64.7)	9 (39.1)	6 (100.0)	42 (66.7)	9 (90.0)
Itching	22 (21.6)	9 (39.1)	0(0.0)	11 (17.6)	2 (20.0)
Arthralgias	11 (10.8)	3 (13.0)	2 (33.3)	5 (7.9)	1 (10.0)
Headache	23 (22.5)	6 (26.1)	3 (50.0)	12 (19.1)	2 (20.0)
Abdominal pain	13 (12.7)	2 (8.7)	1 (16.7)	9 (14.3)	1 (10.0)
Co-infection	20 (19.6)	4 (17.4)	3 (50.00)	9 (14.3)	4 (40.0)

* Patients with other types of rash are not shown.

## Data Availability

Data are available from the corresponding author upon reasonable request.

## Abbreviations

ALT	Alanine aminotransferase
B19V	Human parvovirus B19
CRP	C-reactive protein
EI	Erythema infectiosum
Hgb	Hemoglobin
Ig	Immunoglobulin
PCR	Polymerase chain reaction
EEA	European Economic Area
ECDC	European Center for Disease Prevention and Control
EU	European Union
NPI	Non-pharmaceutical interventions
PAPPE	Parvovirus B19-associated purpuric-petechial eruption
PPGSS	Papular purpuric gloves and socks syndrome
UHID	University Hospital for Infectious Diseases
